# Volatile Compounds of Lemon and Grapefruit IntegroPectin

**DOI:** 10.3390/molecules26010051

**Published:** 2020-12-24

**Authors:** Antonino Scurria, Marzia Sciortino, Alessandro Presentato, Claudia Lino, Elena Piacenza, Lorenzo Albanese, Federica Zabini, Francesco Meneguzzo, Domenico Nuzzo, Mario Pagliaro, Delia Francesca Chillura Martino, Rosa Alduina, Giuseppe Avellone, Rosaria Ciriminna

**Affiliations:** 1 Istituto per lo Studio dei Materiali Nanostrutturati, CNR, Via U. La Malfa 153, 90146 Palermo, Italy; antonino.scurria@ismn.cnr.it (A.S.); claudia.lino@ismn.cnr.it (C.L.); mario.pagliaro@cnr.it (M.P.); 2 Dipartimento di Scienze e Tecnologie Biologiche Chimiche e Farmaceutiche, Università di Palermo, Via Archirafi 32, 90123 Palermo, Italy; marziasciortino@gmail.com; 3 Dipartimento di Scienze e Tecnologie Biologiche Chimiche e Farmaceutiche, Università di Palermo, Via le delle Scienze, 90128 Palermo, Italy; alessandro.presentato@unipa.it (A.P.); elena.piacenza91@gmail.com (E.P.); delia.chilluramartino@unipa.it (D.F.C.M.); valeria.alduina@unipa.it (R.A.); 4 Istituto per la Bioeconomia, CNR, Via Madonna del Piano 10, 50019 Sesto Fiorentino, Italy; lorenzo.albanese@cnr.it (L.A.); federica.zabini@cnr.it (F.Z.); francesco.meneguzzo@cnr.it (F.M.); 5 Istituto per la Ricerca e l’Innovazione Biomedica, CNR, Via U. La Malfa 153, 90146 Palermo, Italy; domenico.nuzzo@cnr.it

**Keywords:** IntegroPectin, lemon, grapefruit, pectin, waste citrus peel, α-terpineol, hydrodynamic cavitation, circular economy

## Abstract

An HS-SPME GC-MS analysis of the volatile compounds adsorbed at the outer surface of lemon and grapefruit pectins obtained via the hydrodynamic cavitation of industrial waste streams of lemon and grapefruit peels in water suggests important new findings *en route* to understanding the powerful and broad biological activity of these new pectic materials. In agreement with the ultralow degree of esterification of these pectins, the high amount of highly bioactive α-terpineol and terpinen-4-ol points to limonene (and linalool) decomposition catalyzed by residual citric acid in the citrus waste peel residue of the juice industrial production.

## 1. Introduction

Industrially extracted from dried lemon (and also orange and lime) peel and in a far lesser amount from apple pomace, pectin is a versatile hydrocolloid that is in high and rapidly increasing demand from the food and pharmaceutical industries [1]. The extraction makes use of diluted mineral or oxalic acid in hot water and employs isopropyl alcohol to precipitate the polysaccharide after the extraction and extensive polymer degradation. Plentiful research has been devoted in the last decade to developing green extraction methods for pectin. Selected achievements include microwave-assisted extraction in water only [2], acoustic cavitation [3], hydro-distillation driven by concentrated solar power [4], enzymatic extraction at acidic pHs [5], and controlled hydrodynamic cavitation (HC) in water only [6]. Lately called “the enabling technology of the bioeconomy” due to its low capital and operational cost, eco-friendly nature, and ease of scaling up [7], HC is increasingly used in the extraction of natural products [8].

Dubbed “IntegroPectin”, citrus pectin extracted from the wet peel of the main citrus fruits residue of the juice production process using HC largely differs from the conventional citrus pectin extracted from dried citrus peel using acid in hot water. Orange IntegroPectin, for instance, has a low degree of esterification (17%) and contains plentiful amounts of co-extracted citrus flavonoids and terpenes [6]. The potential antiviral activity of the main orange flavonoids hesperidin and naringin, for instance, raised interest in HC as an efficient and highly scalable flavonoid production method for the treatment of COVID-19 [9].

Lemon IntegroPectin—namely, citrus pectin extracted from wet industrial waste lemon peel using HC—has exceptional antioxidant [10] and good antibacterial [11] properties, whereas grapefruit IntegroPectin is a broad-spectrum bactericidal agent currently investigated as a natural antimicrobial likely to drive no antimicrobial resistance [12]. Recently named “a universal medicine” in light of its multiple health-beneficial properties (immunomodulating, anticarcinogenic, and antimetastatic properties; anti-inflammatory activity; lowering cholesterol and triglyceride in the blood serum; normalizing glucose metabolism; removing toxins including radionuclides from the body; protecting the gastrointestinal tract) [13], pectin is also a broad-spectrum antibacterial whose antimicrobial properties have been lately rediscovered [14]. It is therefore important, in sight of the medical applications of these new citrus pectins collectively called IntegroPectin, that lemon IntegroPectin is highly cytoprotective, preventing the oxidative degradation of human pulmonary cells from oxidizing agents even at high concentrations of a strong oxidizer such as H_2_O_2_ [10].

The main aim of the study was to analyze the volatile compounds in both lemon and grapefruit IntegroPectin in order to identify the main terpene components of these new and highly bioactive materials, after having identified at least in part the main structural features of the pectic polymers comprising orange [6] and lemon and grapefruit [12]. Indeed, we ascribed the high antimicrobial activity of lemon and grapefruit IntegroPectin to the synergistic action of the chemical structure of citrus pectin extracted using cavitation, combined with the action of the citrus terpenes and flavonoids adsorbed across the pectic polymer obtained upon the lyophilization of the aqueous extract [12]. We briefly remind the reader that no terpenes or flavonoids are found in commercial citrus pectins, as these secondary metabolites are completely removed during the hydrothermal extraction from dried citrus (lemon, orange, and lime) peel using mineral (or oxalic) acid followed by extensive refining and the precipitation of the largely degraded pectic polymer with isopropyl alcohol [1].

The outcomes of this investigation using the headspace solid-phase microextraction (HS-SPME) technique combined with gas chromatography-mass spectrometry (GC-MS) indirectly provide molecular insight into the HC-assisted extraction of both terpenes and pectin from cells comprising waste citrus peel (exo-, meso-, and endocarp) obtained by citrus juice production lines. 

## 2. Results and Discussion

**Figure 1:**
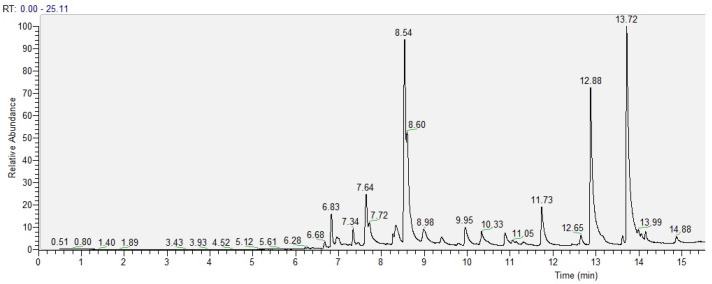
GC chromatogram of the volatiles in lemon IntegroPectin.

A total of 15 volatile compounds were identified in the case of lemon IntegroPectin (Figure 1 and Table 1).

A highly bioactive molecule with antioxidant, anticancer (anti-proliferative and cytostatic), anticonvulsant, antiulcer, antihypertensive, anti-inflammatory, and analgesic (anti-nociceptive) properties [15], α-terpineol is the most abundant component amid the volatiles found in lemon IntegroPectin. The second most abundant terpene is terpinen-4-ol (21.85%, entry 11 in Table 1), a potent bactericidal [16] and antifungal [17] agent capable of killing *Staphylococcus aureus* strains and inhibiting azole-resistant human pathogenic *Candida* species. As the main component of tea tree essential oil, terpinen-4-ol was found in a randomized trial to provide a safe and well-tolerated regimen for the eradication of methicillin-resistant *S. aureus* carriage in hospitalized patients [18]. 

2-methyl-1-butanol (entry 4 in Table 1) and 3-methyl-1-butanol (entry 5) are key components imparting fragrance to grapefruit juice [19] but have been so far unreported in the case of lemon essential oil or juice, thereby indicating the modification of certain volatile molecules during HC-based extraction (see below). Similarly, isoamyl acetate (entry 2 in Table 1) was previously found in orange essential oil [20]. 

**Figure 2:**
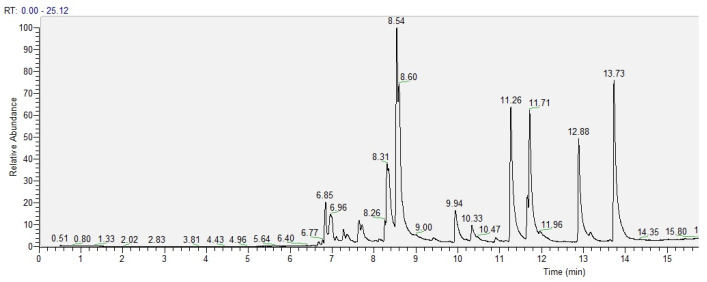
GC chromatogram of the volatiles in grapefruit IntegroPectin.

In the case of grapefruit IntegroPectin, 11 volatile compounds were identified (Figure 2 and Table 2).

Besides a relatively higher amount of limonene (11.8% vs. 3.39%), the main difference with lemon IntegroPectin is the presence of higher amount of linalool (12% vs. 4.95%) as well as of substantial amounts (16%) of linalool oxide (predominantly the *cis* isomer, entry 7 in Table 2), which is absent in lemon IntegroPectin. Linalool oxide isomers are well known components of grapefruit essential oil [21].

Due to an ample and diversified spectrum of biological properties, linalool is one of the most widely studied odorant molecules [22]. Found in the essential oils (EOs) of over 200 plants including citrus, linalool exhibits antimicrobial activity against different microorganisms (*Staphylococcus aureus*, *Bacillus subtilis*, *Escherichia coli*, *Pasteurella multocida*), particularly against Gram-positive bacteria, showing activity levels comparable to those of Amoxicillin [23]. Though far less studied due to its relatively minor abundance in EOs, linalool oxide has been recently shown to have (in experiments with mice) even more powerful anticonvulsant and antinociceptive activities than linalool [24].

**Figure 3:**
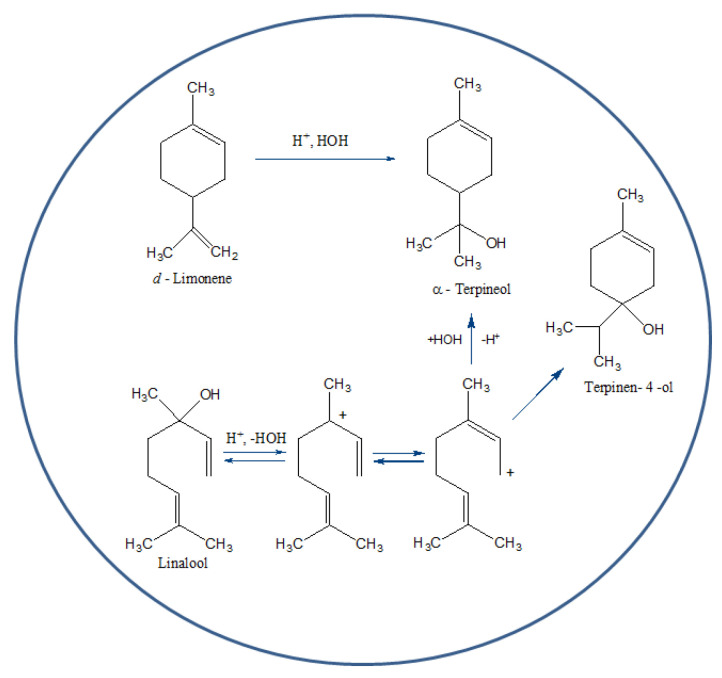
Molecular pathway from linalool and limonene to α-terpineol in the hydrodynamic cavitation microbubbles (adapted from Ref. [27]).

The low amount of limonene, by far the most abundant terpene in the peel of both lemon [25] and grapefruit [26], points to the formation of α-terpineol and terpinen-4-ol from their precursors *d*-limonene and linalool in the cavitation microbubbles, in accordance with what happens in orange [27] and mandarin [28] juices (rich in citric acid) after storage. Following fast protonation likely taking place in the cavitation microbubbles (Figure 3), both linalool and limonene are readily converted into α-terpineol and terpinen-4-ol, with linalool being even more reactive than limonene [27].

The protons needed to drive the reactions displayed in Figure 3 originate from citric acid, a tricarboxylic acid particularly abundant in the lemon pulp from which it was commercially extracted until the introduction of the sugar fermentation process over *Aspergillus niger* [29]. Indeed the industrial waste citrus peel (exo-, meso-, and endocarp) used for the production of orange, lemon, and grapefruit IntegroPectin obtained by citrus juice production lines contains not only the outer skin (exocarp) and the peel (exo- and mesocarp), but also plentiful endocarp residues. Furthermore, experiments with Venturi-shaped HC reactors similar to the reactor used to manufacture the analyzed samples of IntegroPectin clearly showed that hydrophobic molecules (such as terpenes) tend to migrate to the cavity–water interface [30], increasing their concentration close to the water molecules dissolving the H_3_O^+^ ions produced by citric acid hydrolysis, with the extreme local pressures and temperatures created by the collapsing cavities enhancing the reaction rate. This effect is likely to provide a further contribution to the protonation of linalool and limonene and to the observed formation of a terpene nanoemulsion in the HC-derived aqueous extract [6].

Based on this hypothesis, one would expect that the degree of the esterification of lemon, grapefruit, and orange pectin would decrease in the order lemon < grapefruit < orange due to the action of citric acid that is far more abundant in the pulp of lemon (lemon juice typically has a 60 g L^−1^ citric acid concentration vs. 19 g L^−1^ in grapefruit and 11 g L^−1^ in sweet orange [31]). Indeed, this is exactly what is observed, with the ultralow DE (degree of esterification) for lemon IntegroPectin (8%) being lower than that of grapefruit (14%) [12], which is in turn lower than that of orange (17%) [6].

Catalyzed by protons, the de-esterification reaction of pectin through which the methoxyl groups in natural citrus pectin (which is an high-methoxyl pectin) are removed is accelerated in the cavitation bubbles, as discovered in 1974 when studying the acid-catalyzed hydrolysis of methyl acetate in water under acoustic cavitation conditions, when the reaction rate increased up to 9.6% due the “tremendous local pressure and temperature increasing the vibrating motion of the reacting molecules” [32].

The first indirect evidence of limonene conversion during the HC-assisted extraction of waste orange peel was observed by measuring its concentration, when after only 10 min of hydrocavitation the concentration of limonene in the liquid phase decreased by 80% [6]. In this type of extraction, all the terpenes retrieved from the waste citrus peel, including those with little or no solubility in water, do not separate from the water phase, such as in the case of the microwave-assisted hydrodistillation or hydroduffusion and the gravity extraction of waste orange peel and waste lemon peel in water [2].

In brief, the good emulsifying and emulsion-stabilizing properties of pectin [33] are significantly enhanced under hydrodynamic cavitation conditions. As a result, the solutions obtained from the HC-assisted extraction of waste orange, lemon, and grapefruit peels do not separate into a water and an oily phase even after more than 2 years of storage at room temperature, retaining their pleasant odor due to the odorant molecules stabilized against oxidation by the powerful antioxidant activity of the co-extracted citrus flavonoids.

Finally, further proof that the acid-catalyzed molecular degradation of certain citrus terpenes occurs during the HC-based extraction is given by the presence in the lemon IntegroPectin of 2,4-cyclohexadiene-1-methanol, α,α-4-trimethyl (*p*-mentha-1,5-dien-8-ol, entry 15 in Table 1), as well as of 1-hexanol in the grapefruit IntegroPectin (entry 5 in Table 2). The former compound is one of the degradation products of citral (a key aldehyde mixture of neral and geranial) found in earlier studies dealing with the degradation of citral in carbonated orange juice beverages [34]; the latter is likely to be the degradation compound of 3-mercapto-1-hexanol, a sulfur volatile identified in grapefruit juice [35].

## 3. Material and Methods

The GC-MS analysis of the volatile compounds released in the headspace of a closed vial upon their solid phase microextraction (SPME) using a thin polymer coating fixed to the solid surface of a fiber [36] enables the direct identification of the volatile compounds by comparison with the mass spectra of authentic standards. The technique, for example, has been widely used to identify volatiles in the fragrances of lemon cultivars [37]. The analytical conditions used in the present study appear in Table 3.

### 3.1. Materials

Dried samples of lemon and grapefruit IntegroPectin were obtained as previously reported [12]. The divinylbenzene/carboxen/polydimethylsiloxane (DVB/CAR/PDMS, 50/30 μm) fiber was obtained from Supelco (Bellefonte, PA, USA).

### 3.2. Headspace SPME Sampling

**Figure 4:**
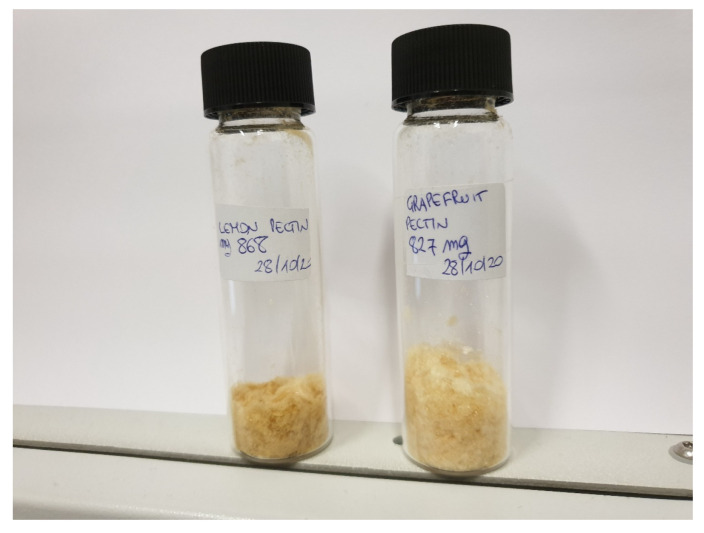
The lemon (**left**) and grapefruit (**right**) IntegroPectin samples analyzed for the volatile content.

Lemon and grapefruit IntegroPectin samples were left in closed vials (Figure 4) at room temperature to equilibrate for 24 h before sampling. The extraction of volatile components accumulated in the headspace took place by injecting the coated fiber in the headspace and leaving the fiber to adsorb the volatiles for 2 min, after which the fiber was withdrawn from the vial and inserted in the hot (260 °C) GC injector.

In order to avoid carry over effects or artifact formation, blank runs were carried out every three analyses. Prior to use and between consecutive analyses, the fiber was conditioned in a GC injector at 260 °C for 3 h to remove contaminants. Nonetheless, siloxane contaminants most likely derived from the injector led to the appearance of interference siloxane peaks in the chromatogram that were readily identified. The fiber used for sampling both lemon and grapefruit pectin used a divinylbenzene/carboxen/polydimethylsiloxane (DVB/CAR/PDMS, 50 μm the DVB layer and 30 μm the CAR/PDMS layer) coating suitable for analyte group flavors (volatiles and semivolatiles). In order to assess the best extraction time for this coating, several preliminary tests at increasing extraction times (2, 5, and 15 min) were investigated (data not shown). The 2 min microextraction time was identified as optimal to reproduce the extraction procedure.

### 3.3. Gas Chromatography-Mass Spectrometry

An ISQ LT single quadrupole mass spectrometer (Thermo Fisher Scientific, Waltham, MA, USA) next to a Trace 1310 gas chromatograph (Thermo Fisher Scientific, Waltham, MA, USA) equipped with a Trace TR-FAME, 100 m × 0.25 mm I.D., 0.20 μm (Thermo Fisher Scientific, Waltham, MA, USA) was used for the GC-MS analyses. The oven temperature program was from 50 °C (hold 0 min) at 10 °C/min to 250 °C, then held for 5 min. The carrier gas was ultrapure helium (He ≥ 99.9999%, ALPHAGAZ 2, Air Liquide Italia Service, Milan, Italy) flown at a 1 mL/min rate. Injection took place in splitless mode at 260 °C. The mass analysis was carried out under conditions of electronic ionization (EI) with an ionization energy of 70 eV. The mass spectra were recorded in full scan in a mass range ranging from 50 to 500 *m/z*. The attribution of compounds corresponding to the chromatographic peaks was performed by comparing the corresponding mass spectra with those of the NIST 11.0 and Wiley 7.0 mass spectrum libraries, verifying the fragmentation through a careful study of the EI spectra.

## 4. Conclusions

The HS-SPME-GC-MS analysis of the volatile compounds of lemon and grapefruit pectins obtained via the hydrodynamic cavitation of lemon and grapefruit peel residue of juice extraction suggests important new findings en route to understanding the high biological activity of these new pectic materials and the hydrodynamic cavitation process applied to wet (i.e., non-dried) waste citrus peel.

The high amount of the highly bioactive terpenoids α-terpineol and terpinen-4-ol points to the conversion of limonene (and linalool) upon protonation and hydration, a process catalyzed by the citric acid present in the endocarp fraction of waste citrus peel and further accelerated under cavitation conditions. This hypothesis also explains the ultralow degree of the esterification of lemon (DE = 8%), grapefruit (DE = 14%), and orange (DE = 17%) IntegroPectin polymers, opening the route to new medical and biotechnological applications of these citrus pectins useful in the nutraceutical, pharmaceutical, food, and personal care/cosmetic industries. For example, scholars in Brazil recently discovered the bactericidal activity of α-terpineol against *S. aureus* strains, interrupting cell division and altering the “grape bunch” morphology typical of *S. aureus* colonies [38]. The findings reported in this study support the similar antimicrobial action of α-terpineol in lemon and grapefruit IntegroPectin [12]. The forthcoming analysis of the flavonoids present in these materials will shed further light on the bioactivity of these new pectic polymers.

## Figures and Tables

**Table 1:** Volatile organic compounds in the lemon IntegroPectin.

Entry	Compound	Apex RT (min)	Area (%)
1	3-Methyl-2-buten-2-ol (prenol)	7.34	1.08
2	1-Butanol, 3-methyl acetate (isoamyl acetate)	8.27	0.73
3	Limonene	8.33	3.39
4	2-Methyl-1-butanol	8.54	17.11
5	3-Methyl-1-butanol	8.6	12.43
6	Eucalyptol	8.98	2.32
7	1-Hexanol	9.95	2.52
8	2-Hexen-1-ol	10.33	2.33
9	6-Hepten-1-ol, 2-methyl	10.88	1.52
10	α-Linalool	11.73	4.94
11	Terpinen-4-ol	12.88	21.85
12	α-Citral	13.62	0.58
13	α-Terpineol	13.72	27.42
14	Safranal	14.15	1.11
15	2,4-Cyclohexadiene-1-methanol, α,α-4-trimethyl	14.88	0.65

**Table 2:** Volatile organic compounds in grapefruit IntegroPectin.

Entry	Compound	RT (min)	%Area
1	1-Butanol, 3-methyl-acetate (isoamyl acetate)	8.26	0.67
2	Limonene	8.31	11.77
3	2-Methyl-1-butanol	8.54	12.91
4	3-Methyl-1-butanol	8.6	15.17
5	1-Hexanol	9.94	3.24
6	3-Hexen-1-ol	10.33	1.28
7	*cis*-Linalool oxide	11.26	14.4
8	*trans*-Linalool oxide	11.66	1.76
9	α-Linalool	11.71	12.01
10	Terpinen-4-ol	12.88	9.37
11	α-Terpineol	13.73	17.4

**Table 3:** Conditions for the analysis.

Sample/Matrix:	0.827 g Freeze-Dried, Ground Grapefruit IntegroPectin; 0.868 g Freeze-Dried, Ground Lemon IntegroPectin
SPME fiber:	50/30 μm DVB/CAR/PDMS
Sample equilibration:	24 h, room temperature
Extraction:	2 min, headspace, room temperature
Column:	TRACE TR-FAME, 100 m × 0.25 mm I.D., 0.20 μm
Oven:	50 °C (0 min), 10 °C/min to 250 °C (5 min)
Injection T:	260 °C
Detector:	DynaMax XR with off-axis dynode, discrete dynode electron multiplier and electrometer, linear range of > 10^7^
Scan range:	full scan, *m*/*z* 50–500
Carrier gas:	He 99.9999%, 1 mL/min constant flow

## Data Availability

All data available by contacting the corresponding authors.

## Sample Availability

Not available.
